# Everybody's Page

**Published:** 1889-02-02

**Authors:** 


### Everybody's Page.

The official report states that there are 135,000 lepers in India, but considering how imperfect Indian statistics still are, it seems possible that they number as many as 250,000.

The following prescription is said to relieve chilblains: Half an ounce of ammonium chloride, dissolved in half a-pint of cider vinegar, and applied frequently.

Dr. Charcot is said to have recently stated it as his opinion that medical women are useful only for their own sex and for children. Well, this gives them a pretty wide field, and, as a matter of fact, they have never sought any other.

Even in Europe death from snake-bite is not unknown. A doctor in La Vendée has been called to aid in 321 cases of poisoning by vipers during the last six years, of which 62 resulted fatally.

Dr. W. J. Collins, who has made a series of experiments on the subject, says that tight lacing lessens the flow of bile —at least, in rabbits. He evidently thinks that there is very little chance of convincing the average woman that she is like a rabbit in this respect.

Auxiliary public schools for feeble-minded children have been established in several of the more important towns in Germany. The test for admission is incapacity to follow the instruction given at the national schools, after a trial lasting at least two years.

Among the expenses of Charles II. are set down the following items: "For 3,685 ribbons distributed with the Royal touch, to cure King's evil, £107; to Mr. Pears, for dissecting a body before the King, £5 1 shilling; to Mr. Knight, for bleeding the King, £10 10 shillings; for a receipt for chocolate, £227."

It is a misfortune to be ill; it is often a blunder; in some places it is a crime. Two rich Americans fell ill of slow fever last summer at Hamburg-les-Bains. When they recovered, their landlord demanded two thousand marks for damages, presumably for making his house unpopular, and, after consulting a lawyer, they had to pay.

Is this a true story? A German surgeon called on Mr. Lawson Tait and asked him what was the secret of his great success in the severest of operations. Mr. Tait glanced at his visitor's hands, and at first evaded answering the question, but at last, on being pressed, said, "I always give the greatest attention to the care of my nails." The German looked at his own nails and turned away. He asked no more questions.

For those who lead a sedentary life, exercise is very necessary, but if they are employed in any intellectual labour their recreation should be of a nature to make few demands on their brain power. For this reason, fencing, gymnastics, riding, and the like may sometimes be unsuited to either the overworked schoolboy or the student of full age, who should in that case try to find exercises which are so simple in character that the action becomes almost mechanical.

Dr. J. M. Nilsen, of New York, having performed a very severe and difficult abdominal operation on a woman, has to confess that she "became demoralised by the unwonted care she had received in hospital, and by the help extended to her after she left, for, although she improved steadily in appearance, she refused even to try to work up to the time of her last visit, about eighteen months after the operation." She evidently appreciated to the full the credit she brought to the operator, and thought that the science of gynæcology owed her a perpetual pension.

In France farm and garden work is regarded as the best employment for most deaf mutes. M. Rivière lately published a manual of gardening and agriculture adapted to their use, which is very much a summary of the practical instructions given by the head gardener to the pupils of the National Institution for Deaf Mutes, in Paris. This institution has a large garden of its own, and, in addition to working in this, the pupils are allowed to assist in the public gardens of the Luxembourg, which is near the school.

The Chinese live in houses where the supply of air is so limited that no European could endure the vitiated atmosphere; yet they are a very healthy nation. This is probably due to the fact that their food is invariably simple, clean, and thoroughly well cooked. Meat, potatoes, and rice, are all boiled together. When cooked the mixture is put into small bowls, and as it is eaten with tiny chopsticks, it is impossible to try the mouth or stomach by scalding them with a quantity of very hot food. Moreover, they rarely drink water if they can get tea, either hot or cold.

A recent French orator, Dr. Charles Levieux, has accredited Moses with being the founder of hospitals. The children of Israel, he argues, having suffered under the yoke of the Egyptians, would readily learn and practise the duty of charity to the unfortunate and the oppressed, when it was inculcated by their great law-giver. As a matter of fact, however, institutions akin to hospitals and dispensaries existed in Egypt before the time of Moses, and we can hardly infer that the Jews showed much interest in hospitals from the solitary allusion to a house of isolation for lepers that is found in Holy Writ.

What next in the way of exhibitions? New ideas are difficult to get hold of, but the firm of Messrs. Pears has hit on something original and attractive. Some time in the coming season they are going to hold a Beauty Show. Fair women from east and west, north and south, are to be invited to enter into competition, and the thirty loveliest are to show themselves, clad in evening dress, in a hall in some easily accessible part of London. As the prizes are valuable —the first amounting to about £200--and the fair exhibits are to have all expenses paid during their visit to London, there is no doubt that there will be plenty of competitors.

Charity has always been an integral part of the Buddhist creed. We learn from Chinese history that under Buddhist influence an asylum for the sick poor was established at Nankin. Soon afterwards an asylum for the very old and the very young was established, and about the same time there was an infirmary at Toyang, the capital of North China. When, in the ninth century, the Buddhist monasteries were suppressed, the Imperial edict which destroyed them ordained that the buildings and lands set apart for the care of the sick poor throughout the empire should be retained for that purpose, and that proper persons should be appointed to take care of the inmates.

Even in would-be ideal republics sanitary blunders will arise. On the "9th of Thermidor, in the 3rd year of the Republic," the health officers laid a petition before the "citizens administrators," stating that an epidemic had broken out among "the prisoners called Chorians." These brave fellows, who had fought in Brittany on the Royalist side, were imprisoned, to the number of 3,600, in an old Capuchin convent. Bad air and bad food developed disease, which infected the surrounding population. The "citizens administrators" had got rid of king and kingdom, but the conquered Chorians were spreading among the citizens of the Republic an epidemic of typhus fever, which was a worse tyrant still.

